# A retrospective analysis of respiratory virus transmission before and during the COVID-19 pandemic in Pune the western region of India

**DOI:** 10.3389/fpubh.2022.936634

**Published:** 2022-09-08

**Authors:** Sumit Bhardwaj, Manohar Lal Choudhary, Sheetal Jadhav, Veena Vipat, Rohan Ghuge, Sonali Salvi, Rajesh Kulkarni, Aarti Kinikar, Vikram Padbidri, Sanjay Bafna, Ashish Bavdekare, Pradeep D'costa, Nilesh Gujar, Varsha Potdar

**Affiliations:** ^1^Influenza, National Institute of Virology (ICMR), Pune, India; ^2^B. J. Medical College and Sassoon Hospital, Pune, India; ^3^Microbiology, Jehangir Hospital, Pune, India; ^4^KEM Hospital Research Centre, Pune, India; ^5^Gujar Children Hospital, Pune, India

**Keywords:** COVID-19, influenza, pandemic, non-pharmaceutical interventions, viral respiratory viruses

## Abstract

**Background:**

SARS-CoV-2 was first reported in China in December 2019 and quickly spread across the world. Non-pharmaceutical interventions (NPIs) are the key to control the transmission of respiratory viruses. To stop the spread, NPI is widely recommended and is still followed by most countries.

**Methods:**

At the National Influenza Center of the Indian Council of Medical Research-National Institute of Virology (ICMR-NIV), the surveillance of severe acute respiratory illness and acute respiratory illness cases for influenza and other respiratory viruses is in place. In this study, we analyzed surveillance data on respiratory viruses and/or SARS-CoV-2 testing from January 2017 to December 2021. Multiplex real-time PCR was used to detect the respiratory viruses.

**Results:**

Our findings indicate that during the pandemic, the positivity for influenza A and B, metapneumovirus, parainfluenza virus, respiratory syncytial virus, and human coronavirus declined significantly.

**Conclusion:**

The annual distinct seasonal outbreaks of influenza, RSV, and other respiratory viruses as observed during the pre-COVID-19 period were not observed during the COVID-19 pandemic in years 2020 and 21. Social distancing, lock-downs, and non-pharmaceutical interventions may play an important role in the reduction of respiratory viruses. Understanding the seasonal respiratory virus decline could help public health experts prepare for future respiratory virus pandemics.

## Introduction

In adults, a Human metapneumovirus (hMPV) and human coronaviruses (CoV), as well as adenoviruses (AdV) and parainfluenza viruses (PIV) are all respiratory viruses that contribute to both morbidity and mortality (1, 2), particularly among older and vulnerable adult populations (3). Furthermore, they account for huge economic expenses on a global scale each year (4). Non-pharmaceutical interventions (NPIs) to control the transmission of respiratory viruses are essential since pharmacological treatment options for most of these viruses are limited. SARS-CoV-2 was first reported in China in December 2019 and quickly spread across the country. Governments have adopted various non-pharmacological public health measures, such as mask use, social isolation, and hand hygiene, to try to minimize the spread of SARS-CoV-2. Prevention is critical when dealing with a disease that lacks a particular therapy to reduce morbidity and death. Vaccination has the potential to be a very successful future preventative method (5–9). NPI's are mainly recommended and is still widely recommended by most countries to stop the spread of SARS-CoV-2. According to national recommendations, the general populace used face masks, maintained social distance, performed basic hand hygiene, cleaned surfaces, and ventilated interior spaces (10). During lockdown, religious institutions, schools, and malls were closed, and social and religious gatherings were restricted, resulting in a significant loss of social connections. Even though there is currently widespread agreement regarding their efficacy in containing SARS-CoV-2 (11). Based on data from India's national influenza center surveillance system, which tracks ARIs, particularly influenza, from 2017 to 2021, especially from western region of India, we demonstrate that the restrictions prevented not just the spread of SARS-CoV-2, but also the transmission of other viral diseases as a result of the restrictions.

## Methods

This retrospective study was carried out by the National Influenza Center of the Indian Council of Medical Research-National Institute of Virology (ICMR-NIV). From January 2017 to December 2021, we gathered epidemiological and laboratory diagnosis data on respiratory virus and/or SARS-CoV-2, and analyzed for two periods as pre-pandemic period January 2017–March 2020 and pandemic period April 2020–December 2021. The analyzed data are part of the ongoing surveillance of Influenza and other respiratory viruses. The study center recruited participants from sentinel hospitals and clinics with general medical and pediatric departments from Pune, India. The institutional human ethics committee's approval was obtained. The research was explained to the participants in their local language, and written consent or assent was obtained before enrollment. Acute respiratory infection (ARI) was defined in the case definitions used to enroll participants as cases presenting in the outpatient department (OPD) with an acute onset (within 7 days) of at least two of the following symptoms: fever/feverishness, chills, cough, nasal congestion, shortness of breath, or sore throat. Patients with modified SARI were defined as those who had a history of cough that began during the past seven days and that required overnight hospitalization. SARI was defined in babies < 2 months of age as a physician diagnosis consistent with an acute lower respiratory infection (pneumonia, bronchitis, bronchiolitis, sepsis) necessitating overnight hospitalization. The four sentinel hospitals represented both the public and private sectors. Center recruited 10–20 ARI and SARI patients each week from sentinel hospitals across all age categories. Throughout the research period, participants were recruited *via* convenient sampling. Additional individuals were added during a surge of respiratory illnesses in the community or hospital. Physicians and nurses at all sentinel locations were trained to screen patients using the ARI and SARI case criteria. Each recruited participant's clinical and epidemiological data were documented on a standardized case report form. Clinical samples i.e., nasal/throat swab were collected, transported, and stored in accordance with WHO recommendations (12). Trained staff collected nasal, throat, or nasopharyngeal respiratory specimens (according to age) and transported them to the laboratory within 24 h while maintaining a cold chain. RNA was extracted according to the manufacturer's procedure using a MagMax-96 kit. All specimens were tested for the following viruses using the real-time reverse transcription polymerase chain reaction (rRT-PCR): [influenza A(H1N1) pdm09, A (H3N2)], influenza B [B/Yamagata and B/Victoria] along with the house-keeping RNaseP gene (CDC, WHO), respiratory syncytial virus A and B, metapneumovirus, parainfluenza virus 1,2,3,4, rhinovirus and SARS-CoV-2 (13–15). A one-step reverse transcriptase polymerase chain reaction was used to amplify nucleic acids (qRT-PCR SuperScript TM III kit, Invitrogen, USA). A 25 ul PCR reaction included 10 PM of forward and reverse primers, five PM of TaqMan probe, 12.5 milliliters of 2X buffer, 0.5 milliliters of SuperScriptTM III enzyme, and five milliliters of nucleic acid templates. Thermal cycling conditions were as follows: 30 min at 50°C for reverse transcription, 5 min at 94°C for initial denaturation, 45 cycles of three stages-−15 sec at 94°C, 15 sec at 50°C, and 30 sec at a 55°C incubation step for data collection. Allelic discrimination was used to identify an oseltamivir-resistant A/H1N1pdm virus with the H275Y mutation [Neuraminidase (NA)] in clinical specimens or clinical isolates, using the RT-PCR procedure given by the National Institute of Health, Thailand].

Data were entered into Epi-info 7 (TM), which was compiled weekly. The epidemiological week and seasonality were calculated based on the day of illness onset. Weekly data was then shown as percent positive for each virus over the course of an epi-year (since the beginning of analysis). We used the chi-square test to determine the statistical significance of the proportions. The study duration was divided into pre-pandemic (January 2017 to March 2020) and pandemic (April 2020 to December 2021); post-pandemic was further divided into P1: early stages of pandemic (April 2020 to December 2020) and P2: late stages of pandemic (January 2021 to December 2021).

## Results

**Figure 1:**
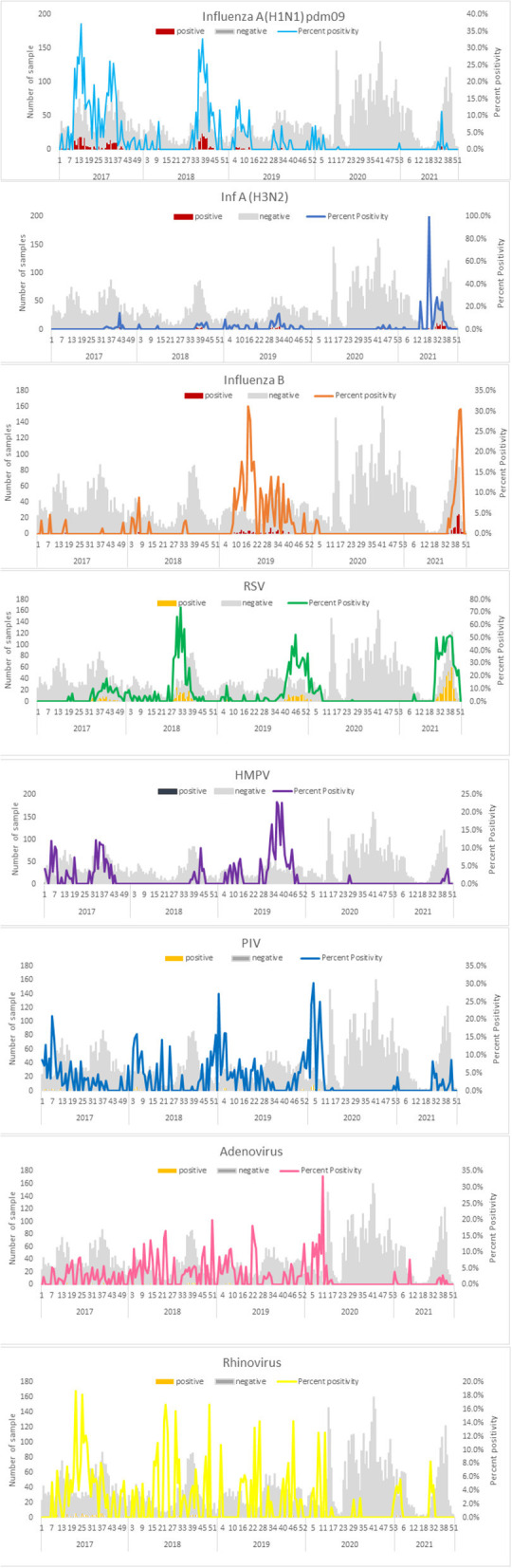
Weekly positive cases of respiratory pathogens in the pandemic period (April 2020–December 2021) in comparison with monthly positive cases in the pre-pandemic period (January 2017–March 2020).

During the research period, 9,613 patients were tested for respiratory viruses and analyzed. 5190 were studied between January 2017 and March 2020; 3206 between April and December 2020; and 1,217 between January and December 2021. Table 1 summarizes the patient demographic characteristics. From March 2020, as the number of COVID-19 infections grew, the number of tests needed by doctors increased proportionately (32/week during the pre-pandemic period vs. 47/week during the pandemic). More importantly, during the early stages of the pandemic (P1), the overall test positivity for non-SARS CoV-2 respiratory viruses was lower than it had been in the previous years (3.3 vs. 33.9%, respectively, p 0.0001). The test positivity increased to 45% in the latter part of 2021. Demographic characteristics indicate that male gender was more common (56.4 vs. 61.7%, p 0.0001) and the median age was higher (19.3 years vs. 41 years, p 0.0001) in the adult population examined during the pandemic era (P1). The rate of positives for each respiratory virus examined throughout the research period is shown in Table 2. The test positivity for influenza influenza A(H1N1) pdm09, influenza A (H3N2), influenza B, HMPV, PIV1-4, RSV A & B, AdV, and rhinovirus significantly decreased during the pandemic, especially in the early stages of the pandemic when government restrictions were more stringent. Children under the age of five were found to have the highest levels of viral positivity both before and during the pandemic. However, when compared to the period before the pandemic, their positivity decreased significantly during the early stages of the pandemic (P1) (18.4% vs. 44.7 %, p 0.0001). When other respiratory virus-positive subjects were stratified by age, the same results were obtained. Similar findings were found when additional respiratory virus-positive participants were stratified by age (Supplementary Table 1). The viral positivity for ARI cases was higher among all viruses compared to SARI cases except for rhino and adenovirus (Supplementary Table 3) Before the pandemic, influenza virus activity was usually at its highest between June and September, with an intersessional surge in February and March (Figure 1). However, during the pandemic period, no influenza activity was observed during the early stages (P1). From August to December 2021, both influenza A and B activity were seen without regard to seasonal patterns. Prior to the pandemic, RSV, HMPV, PIV1-4, and AdV circulated from monsoon to winter, peaking between September and December, while during the pandemic, they were detected only in the latter half of the 2021 (P2), with essentially little activity of positive cases in the early stages of the pandemic (P1). Additionally, certain coinfections were also identified (Table 3) (79/5190 [1.5%] before the pandemic, 35/4427 [0.7%] during the pandemic). However, no correlation of co-infection with clinical outcome was observed. On January 30, 2020, India reported its first COVID-19 case. Immediately following the case's emergence, the government enacted various community-based mitigating strategies, including school closures and social isolation (Supplementary Table 2). Following the index case in India, SARS-CoV-2 has become the primary cause of respiratory infections.

**Table 1:** Characteristics of patients tested for respiratory viruses other than SARSCoV-2 between January 2017 and May 2021.

	**January 2017– March 2020**		**April 2020–December 2020 (P1)**		**Jan 2021–December 2021 (P2)**	
	**Pre–Pandemic**		**Early Stage of Pandemic**		**Later Stage of Pandemic**	
	**No**.	**Percent**	**No**.	**Percent**	**No**.	**Percent**
Sample tested	5190		3206		1217	
Male	2928	56.4%	1977	61.7%	672	55.2%
Mean age (years)	19.3		41		22	
**Age in years**						
<2	1317	25.4%	252	7.9%	375	30.8%
2– <5	799	15.4%	69	2.2%	170	14.0%
5– <15	1007	19.4%	154	4.8%	139	11.4%
15– <45	1190	22.9%	1205	37.6%	260	21.4%
45– <60	401	7.7%	694	21.6%	111	9.1%
>60	476	9.2%	832	26.0%	162	13.3%
**Type of sample**						
ARI	1730	33.3%	830	25.9%	313	25.7%
SARI	3460	66.7%	2376	74.1%	904	74.3%

**Table 2:** Comparison of positivity rates of respiratory viruses other than SARSCoV−2 between January 2017–March 2020 (pre–pandemic period) and April 2020–December 2021 (during pandemic period).

	**January 2017– March 2020 (P1)**		**March 2020–December 2020 (P2)**		**P1 vs P2**	**Jan 2021–December 2021 (P3)**	
	**Pre–Pandemic**		**Early Stage of Pandemic**			**Later Stage of Pandemic**	
	**No**.	**Percent**	**No**.	**Percent**		**No**.	**Percent**
Overall positivity	1708	32.9%	107	3.3%	<0.001	550	45.2%
Influenza A(H1N1) pdm09	422	8.1%	5	0.2%	<0.001	10	0.8%
Inf A (H3N2)	75	1.4%	13	0.4%	<0.001	73	6.0%
Inf B	165	3.2%	3	0.1%	<0.001	93	7.6%
RSV	394	7.6%	18	0.6%	<0.001	332	27.3%
HMPV	170	3.3%	2	0.1%	<0.001	5	0.4%
PIV 1–4	165	3.2%	36	1.1%	<0.001	15	1.2%
Adenovirus	153	2.9%	18	0.6%	<0.001	9	0.7%
Rhinovirus	164	3.2%	12	0.4%	<0.001	13	1.1%
**Positive for Any of the respiratory virus**							
<2 yrs	600	45.6%	47	18.7%	<0.001	273	72.8%
2– <5 yrs	345	43.2%	12	17.4%	<0.001	119	70.0%
5– <15 yrs	267	26.5%	22	14.3%	<0.001	80	57.6%
15– <45 yrs	233	19.6%	16	1.3%	<0.001	38	14.6%
45– <60 yrs	96	23.9%	6	0.9%	<0.001	8	7.2%
>60 yrs	87	18.3%	16	1.9%	<0.001	8	4.9%
**SARS CoV−2**							
<2 yrs	NA	NA	2	0.8%		0	0.0%
2– <5 yrs	NA	NA	5	7.2%		1	0.6%
5– <15 yrs	NA	NA	6	3.9%		4	2.9%
15– <45 yrs	NA	NA	207	17.2%		33	12.7%
45– <60 yrs	NA	NA	150	21.6%		28	25.2%
>60 yrs	NA	NA	153	18.4%		38	23.5%

**Table 3:** Co–infection during the study period.

**January 2017– March 2020**	**H1N1**	**H3N2**	**B Victoria**	**B Yamagata**	**RSV**	**PIV**	**HMPV**	**Adenovirus**	**Rhinovirus**
H1N1 pdm09		0	0	0	11	2	2	1	2	
H3N2			0	0	0	1	0	1	0	
B Victoria				0	1	1	0	1	1	
B Yamagata					1	0	0	0	0	
RSV						7	3	8	15	
PIV							1	4	4	
HMPV								4	3	
Adenovirus									5	
Rhinovirus										
**April 2020–December 2021**	**H1N1**	**H3N2**	**B Victoria**	**B Yamagata**	**RSV**	**PIV**	**HMPV**	**Adenovirus**	**Rhinovirus**	**SARS CoV**−**2**
H1N1 pdm09		1	0	0	1	1	0	0	0	0
H3N2			1	0	7	0	0	0	1	1
B Victoria				0	8	0	0	2	0	0
B Yamagata					0	0	0	0	0	0
RSV						4	0	4	1	0
PIV							0	1	1	0
HMPV								0	0	0
Adenovirus									0	0
Rhinovirus										1
SARS CoV−2										

## Discussion

Our study suggests that the NPI's used to contain the spread of COVID-19, such as social isolation, face mask use, and increased hand hygiene, may also help prevent the spread of other respiratory virus infections that are transmitted in a similar manner to SARSCoV-2. During the early stage (P1) of the COVID-19 pandemic, a significant decline in the prevalence of RSV, influenza A and B, hMPV, and PIV was seen in comparison to previous years. We also noticed a significant decrease in the frequency of hAdV and rhinoviruses, but not as substantially as other respiratory viruses. Notably, our data indicated that the most prevalent seasonal viruses (e.g., hRSV, influenza, PIV, and hMPV) were severely limited during the COVID-19 pandemic, particularly during the early stages. Recent investigations globally have shown similar findings (16–19). This fall in non-SARS-CoV-2 respiratory virus detection is consistent with studies from the United States and the United Kingdom suggesting an early termination to the 2019/2020 influenza season and a decline in other virus detection during the pandemic's early phases (18, 20). Additionally, multiple studies conducted in different countries discovered a considerable decline in influenza virus detection during the early stages of the COVID-19 pandemic (21–25). Other research (20, 22, 26–30) shows that the pandemic interrupted respiratory viral circulation, even though the amount of this impact differed amongst the viruses investigated. We observed that influenza and other respiratory viruses dramatically decreased in frequency during the COVID-19 pandemic. We previously established that these viruses' exhibit seasonality and consecutive seasonal epidemic patterns, but respiratory virus epidemic patterns have changed substantially in the aftermath of the introduction of SARS-CoV-2. Following that, in March 2020, the influenza virus was transmitted at very low levels, with just a few cases recorded over the 2020 season. According to these results, research conducted in the United States of America discovered that positive rates decreased dramatically during the lockdown and remained low during the inter-seasonal circulation (20). We discovered that a decrease in constraints on population movement in the second half of 2021 resulted in an increase in the spread of influenza viruses, hPIV, hRSV, hMPV, and hAdV. Our study group found that males had a higher rate of respiratory virus positivity than females, which is consistent with the literature (31.9 vs 30.5% in the pre-pandemic and 30.2 vs. 26% during the pandemic). A similar observation was made by Jeon et al. in Northern United Arab Emirates (52.8 vs. 50%) (31). According to Rath et al., among hospitalized patients with influenza, men had a greater incidence of positive tests than females, and this was especially true in males (56 vs. 44 %) (32). As shown in US data by Kim et al., the adjusted risk ratio for intensive care unit admission in men is 1.34 times higher than in females (33). These findings suggest that males are more likely than females to be hospitalized for respiratory viruses, which may explain the high male rate in our patient population. Our investigation found few respiratory co-pathogens in COVID-19 individuals. This discovery might have a considerable influence on the diagnostic flow of ARI patients in high-COVID-19 prevalence areas, casting doubt on the necessity to look for viruses other than SARS-CoV-2 right away. Our results support a growing body of literature demonstrating that strict public health interventions, including regional lockdowns, border closures, handwashing, and facemask usage, may greatly reduce the transmission of epidemic respiratory viruses. Despite the continuous identification of SARS-CoV-2, this research is notable for the reported substantial drop in non-SARS-CoV-2 respiratory viruses. This might be because of increased SARSCoV-2's transmissibility compared to other respiratory viruses, which outcompete seasonal respiratory viruses, which are more readily influenced by SARS-CoV-2 transmission mitigation efforts. It is now difficult to say if viral-viral interactions between SARS-CoV-2 and other respiratory viruses are also contributing to the observed drop in circulation. Nickbakhsh et al. hypothesized that viral interference may be achieved by interferon-mediated mechanisms (34). The study's strength comes from the utilization of data from a robust surveillance system that was in operation during the COVID-19 pandemic in 2020, as well as a well-defined case definition. In addition, the data includes hospital surveillance from both the OPD and the IPD. Consequently, this research looked at even the less critical cases that did not need hospitalization. This study may have a number of potential limitations. This is retrospective observational research, so it is impossible to say if the observed decline is due to multilayered public health efforts focused on preventing the spread of SARS-CoV-2 or other unknown causes. In addition, the sample is convenient, which may not reflect the total number of patients that visit the OPD and IPD. To delimit these limitations in future, more structured way of data collection and enumerating the number of cases seeking care for ARI would be important to understand the multipliers for estimate generation.

In conclusion, the annual seasonal outbreak for most non-SARS-CoV-2 respiratory viruses is missing in 2020/21. An unknown number of variables including non-pharmaceutical interventions might be responsible for this drastic drop. Stringent SARS-CoV-2 containment likely reduced the spread of other respiratory viruses. However, the function of SARS CoV-2 in viral displacement and interference is unknown. For public health purpose, understanding how seasonal respiratory viruses decline may prevent future seasonal respiratory virus epidemics and prepare for future respiratory virus pandemics.

## Data availability statement

The original contributions presented in the study are included in the article/Supplementary material, further inquiries can be directed to the corresponding author.

## Ethics statement

The studies involving human participants were reviewed and approved by ICMR-NIV, Pune. Written informed consent to participate in this study was provided by the participants' legal guardian/next of kin.

## Author contributions

SB: conceptualization, data curation, formal analysis, writing—original draft, writing—review, and editing. MC: conceptualization, validation, data curation, and administration. SJ: investigation and data curation. VV: investigation. RG: formal analysis. SS, RK, AK, ViP, SB, AB, PD'c, and NG: investigation. VaP: conceptualization, funding acquisition, methodology, project administration, resources, supervision, writing—review, and editing. All authors contributed to the article and approved the submitted version.

## Conflict of interest

The authors declare that the research was conducted in the absence of any commercial or financial relationships that could be construed as a potential conflict of interest.

## Publisher's note

All claims expressed in this article are solely those of the authors and do not necessarily represent those of their affiliated organizations, or those of the publisher, the editors and the reviewers. Any product that may be evaluated in this article, or claim that may be made by its manufacturer, is not guaranteed or endorsed by the publisher.

## References

[1] James SL Abate D Abate KH Abay SM Abbafati C Abbasi N. Global, regional, and national incidence, prevalence, and years lived with disability for 354 diseases and injuries for 195 countries and territories, 1990–2017: a systematic analysis for the Global Burden of Disease Study 2017. Lancet. (2018) 392:1789–858. 10.1016/S0140-6736(18)32279-7 30496104 PMC6227754

[2] Roth GA Abate D Abate KH Abay SM Abbafati C Abbasi N. Global, regional, and national age sex-specific mortality for 282 causes of death in 195 countries and territories, 1980–2017: a systematic analysis for the Global Burden of disease Study 2017. Lancet. (2018) 392:1736–88. 10.1016/S0140-6736(18)32203-7 30496103 PMC6227606

[3] Blackburn R Zhao H Pebody R Hayward A Warren-Gash C. Laboratory-confirmed respiratory infections as predictors of hospital admission for myocardial infarction and stroke: Time series analysis of English data for 2004–2015. Clin Infect Dis. (2018) 67:8–17. 10.1093/cid/cix1144 29324996 PMC6005111

[4] Fendrick A Monto AS Nightengale B Sarnes M. The economic burden of non-influenza-related viral respiratory tract infection in the United States. Arch Intern Med. (2003) 163:487–94. 10.1001/archinte.163.4.487 12588210

[5] Dai L Gao GF. Viral targets for vaccines against COVID-19. Nat Rev Immunol. (2021) 21:73–82. 10.1038/s41577-020-00480-0 33340022 PMC7747004

[6] Polack FP Thomas SJ Kitchin N Absalon J Gurtman A Lockhart S. Safety and efficacy of the BNT162b2 mRNA Covid-19 vaccine. N Engl J Med. (2020) 383:2603–15. 10.1056/NEJMoa2034577 33301246 PMC7745181

[7] Ramasamy MN Minassian AM Ewer KJ Flaxman AL Folegatti PM Owens DR. Safety and immunogenicity of ChAdOx1 nCoV-19 vaccine administered in a prime-boost regimen in young and old adults (COV002): a single-blind, randomised, controlled, phase 2/3 trial. Lancet. (2021) 396:1979–93. 10.1016/S0140-6736(20)32466-1 33220855 PMC7674972

[8] Wang Z Schmidt F Weisblum Y Muecksch F Barnes CO Finkin S. mRNA vaccine-elicited antibodies to SARS-CoV-2 and circulating variants. Nature. (2021) 592:616–22. 10.1038/s41586-021-03324-6 33567448 PMC8503938

[9] Fiocchi A Jensen-Jarolim E. SARS-CoV-2, can you be over it. World Allergy Organ J. (2021) 14:100514. 10.1016/j.waojou.2021.100514 33552379 PMC7846213

[10] CIRCULARS FOR COVID-19. Available online at: https://www.mha.gov.in/sites/default/files/PR_ConsolidatedGuidelinesofMHA_28032020_0.pdf (accessed August 26, 2022).

[11] Jefferson T Del Mar CB Dooley L Ferroni E Al-Ansary LA Bawazeer GA. Physical interventions to interrupt or reduce the spread of respiratory viruses. Cochrane Database Syst Rev. (2020) 11:CD006207. 10.1002/14651858.CD006207.pub5 33215698 PMC8094623

[12] Manual, W. H. O. for the Laboratory Diagnosis and Virological Surveillance of Influenza Available online at: https://apps.who.int/iris/handle/10665/44518 (accessed August 26, 2022).

[13] CDC protocol of realtime RTPCR for swine influenza A(H1N1) 2009. Available online at: https://www.cdc.gov/h1n1flu/lab/ (accessed August 26, 2022).

[14] Koul PA Mir H Saha S Chadha MS Potdar V Widdowson MA. Respiratory viruses in returning Hajj & Umrah pilgrims with acute respiratory illness in 2014-2015. Indian J Med Res. (2018) 148:329–33. 10.4103/ijmr.IJMR_890_17 30425224 PMC6251276

[15] Potdar V Choudhary ML Bhardwaj S Ghuge R Sugunan AP Gurav Y. Respiratory virus detection among the overseas returnees during the early phase of COVID-19 pandemic in India. Indian J Med Res. (2020) 151:486–9. 10.4103/ijmr.IJMR_638_20 32474556 PMC7530434

[16] Redlberger-Fritz M Kundi M Aberle SW Puchhammer-Stöckl E. Significant impact of nationwide SARS-CoV-2 lockdown measures on the circulation of other respiratory virus infections in Austria. J Clin Virol. (2021) 137:104795. 10.1016/j.jcv.2021.104795 33761423 PMC7962988

[17] Tang JW Bialasiewicz S Dwyer DE Dilcher M Tellier R Taylor J. Where have all the viruses gone? disappearance of seasonal respiratory viruses during the COVID-19 pandemic. J Med Virol. (2021) 93:4099–101. 10.1002/jmv.26964 33760278 PMC8250511

[18] Poole S Brendish NJ Clark TW. SARS-CoV-2 has displaced other seasonal respiratory viruses: results from a prospective cohort study. J Infect. (2020) 81:966–72. 10.1016/j.jinf.2020.11.010 33207254 PMC7666810

[19] Maharaj AS Parker J Hopkins JP Gournis E Bogoch II Rader B. The effect of seasonal respiratory virus transmission on syndromic surveillance for COVID-19 in Ontario, Canada. Lancet Infect Dis. (2021) 21:593–4. 10.1016/S1473-3099(21)00151-1 33773620 PMC7993926

[20] Olsen SJ Azziz-Baumgartner E Budd AP Brammer L Sullivan S Pineda RF. Decreased influenza activ- ity during the COVID-19 pandemic—United States, Australia, Chile, and South Africa, 2020. Morb Mortal Wkly Rep. (2020) 20:3681–5. 10.1111/ajt.16381 33264506 PMC7753605

[21] Suntronwong N Thongpan I Chuchaona W Lestari FB Vichaiwattana P Yorsaeng R. Impact of COVID-19 public health interventions on influenza incidence in Thailand. Pathog Glob Health. (2020) 114:225–7. 10.1080/20477724.2020.1777803 32521210 PMC7480427

[22] Lee H Lee H Song KH. Impact of public health interventions on sea- sonal influenza activity during the COVID-19 Outbreak in Korea. Clin Infect Dis. (2020) 13620, 1–9. 10.1093/cid/ciaa672 32472687 PMC7314207

[23] S-Ch Kuo S-M Shih Chien L-H Hsiung CA. Collateral benefit of COVID-19 control measures on influenza activity, Taiwan. Emerg Infect Dis. (2020) 26:1928–30. 10.3201/eid2608.201192 32339091 PMC7392415

[24] Sakamoto H Ishikane M Ueda P. Seasonal Influenza Activity During the SARS–CoV-2 Outbreak in Japan. JAMA. (2020) 323:1969–71. 10.1001/jama.2020.6173 32275293 PMC7149351

[25] Sunagawa S Iha Y Kinjo T. Disappearance of summer influenza in the Oki- nawa prefecture during the severe acute respiratory syndrome coronavirus 2 (SARS-CoV-2) pandemic. Respir Investig. (2021) 59:149–52. 10.1016/j.resinv.2020.10.010 33246913 PMC7667393

[26] Fricke LM Glöckner S Dreier M Lange B. Impact of non-pharmaceutical interventions targeted at COVID-19 pandemic on influenza burden- a systematic review. J Infect. (2021) 82:1–35. 10.1016/j.jinf.2020.11.039 33278399 PMC9183207

[27] Kim MC Kweon OJ Lim YK Choi SH Chung JW Lee MK. Impact of social distancing on the spread of common respiratory viruses during the coronavirus disease outbreak. PLoS ONE. (2021) 16:e0252963. 10.1371/journal.pone.0252963 34125839 PMC8202938

[28] Park KY Seo S Han J Park JY. Respiratory virus surveillance in Canada during the COVID-19 pandemic: An epidemiological analysis of the effectiveness of pandemic-related public health measures in reducing seasonal respiratory viruses test positivity. PLoS ONE. (2021) 16:e0253451. 10.1371/journal.pone.0253451 34143839 PMC8213179

[29] Rodgers L Sheppard M Smith A Dietz S Jayanthi P Yuan Y. Changes in seasonal respiratory illnesses in the United States during the coronavirus disease 2019 (COVID-19) pandemic. Clin Infect Dis. (2021) 73:S110–7. 10.1093/cid/ciab311 33912902 PMC8135472

[30] Parry F Shah AK Sestovic M Salter S. Precipitous fall in common respiratory viral infections during COVID-19. Open Forum Infect Dis. (2020) 11:ofaa511. 10.1093/ofid/ofaa511 33269297 PMC7665739

[31] Jeon JH Han M Chang HE Park SS Lee JW Ahn YJ. Incidence and seasonality of respiratory viruses causing acute respiratory infections in the Northern United Arab Emirates. J Med Virol. (2019) 91:1378–84. 10.1002/jmv.25464 30900750 PMC7166826

[32] Rath B Conrad T Myles P Alchikh M Ma X Hoppe C. Influenza and other respiratory viruses: standardizing disease severity in surveillance and clinical trials. Expert Rev Anti Infect Ther. (2017) 15:545–68. 10.1080/14787210.2017.1295847 28277820 PMC7103706

[33] Kim L Garg S O'Halloran A Whitaker M Pham H Anderson EJ. Risk factors for intensive care unit admission and in-hospital mortality among hospitalized adults identified through the US coronavirus disease 2019 (COVID-19)-associated hospitalization surveillance network (COVID-NET). Clin Infect Dis. (2021) 72:e206–14. 10.1093/cid/ciaa1012 32674114 PMC7454425

[34] Nickbakhsh S Mair C Matthews L. Virus-virus interactions impact the population dynamics of influenza and the common cold. Proc Natl Acad Sci U S A. (2019) 116:27142–50. 10.1073/pnas.1911083116 31843887 PMC6936719

