# Supplementary material for: A retrospective analysis of respiratory virus transmission before and during the COVID-19 pandemic in Pune the western region of India
Source: Front Public Health. 2022 Sep 8;10:936634. doi: 10.3389/fpubh.2022.936634 (PMC9494283; doi:10.3389/fpubh.2022.936634)
Supplement: Supplementary file 1 [file Data_Sheet_1.doc]

**Supplementary Table 1 Various respiratory positive cases stratified by age.**

|  | **Pre-Pandemic** | | **Early Stage of Pandemic** | | **Later Stage of Pandemic** | |
| --- | --- | --- | --- | --- | --- | --- |
|  | **No.** | **Percent** | **No.** | **Percent** | **No.** | **Percent** |
| **Inf A pdm09 (H1N1)** | | |  |  |  |  |
| **< 2 yrs** | 63 | 1.20% | 0 | 0.00% | 4 | 0.30% |
| **2-<5 yrs** | 71 | 1.40% | 1 | 0.00% | 2 | 0.20% |
| **5-<15 yrs** | 65 | 1.30% | 2 | 0.10% | 3 | 0.20% |
| **15-<45 yrs** | 121 | 2.30% | 2 | 0.10% | 1 | 0.10% |
| **45-<60 yrs** | 61 | 1.20% | 0 | 0.00% | 0 | 0.00% |
| **>60 yrs** | 41 | 0.80% | 0 | 0.00% | 0 | 0.00% |
| **Inf A (H3N2)** | |  |  |  |  |  |
| **< 2 yrs** | 11 | 0.20% | 0 | 0.00% | 13 | 1.10% |
| **2-<5 yrs** | 19 | 0.40% | 2 | 0.10% | 19 | 1.60% |
| **5-<15 yrs** | 21 | 0.40% | 3 | 0.10% | 24 | 2.00% |
| **15-<45 yrs** | 13 | 0.30% | 6 | 0.20% | 10 | 0.80% |
| **45-<60 yrs** | 4 | 0.10% | 1 | 0.00% | 4 | 0.30% |
| **>60 yrs** | 7 | 0.10% | 1 | 0.00% | 3 | 0.20% |
| **INF B** |  |  |  |  |  |  |
| **< 2 yrs** | 18 | 0.30% | 1 | 0.00% | 29 | 2.40% |
| **2-<5 yrs** | 40 | 0.80% | 0 | 0.00% | 17 | 1.40% |
| **5-<15 yrs** | 64 | 1.20% | 2 | 0.10% | 34 | 2.80% |
| **15-<45 yrs** | 27 | 0.50% | 0 | 0.00% | 12 | 1.00% |
| **45-<60 yrs** | 4 | 0.10% | 0 | 0.00% | 0 | 0.00% |
| **>60 yrs** | 13 | 0.30% | 0 | 0.00% | 1 | 0.10% |
| **RSV A &B** | |  |  |  |  |  |
| **< 2 yrs** | 245 | 4.70% | 15 | 0.50% | 219 | 18.00% |
| **2-<5 yrs** | 78 | 1.50% | 1 | 0.00% | 83 | 6.80% |
| **5-<15 yrs** | 31 | 0.60% | 0 | 0.00% | 17 | 1.40% |
| **15-<45 yrs** | 22 | 0.40% | 0 | 0.00% | 9 | 0.70% |
| **45-<60 yrs** | 7 | 0.10% | 1 | 0.00% | 2 | 0.20% |
| **>60 yrs** | 11 | 0.20% | 1 | 0.00% | 2 | 0.20% |
| **HMPV** |  |  |  |  |  |  |
| **< 2 yrs** | 81 | 1.60% | 0 | 0.00% | 4 | 0.30% |
| **2-<5 yrs** | 46 | 0.90% | 0 | 0.00% | 0 | 0.00% |
| **5-<15 yrs** | 20 | 0.40% | 0 | 0.00% | 1 | 0.10% |
| **15-<45 yrs** | 10 | 0.20% | 0 | 0.00% | 0 | 0.00% |
| **45-<60 yrs** | 6 | 0.10% | 0 | 0.00% | 0 | 0.00% |
| **>60 yrs** | 7 | 0.10% | 2 | 0.10% | 0 | 0.00% |
| **PIV 1-4** |  |  |  |  |  |  |
| **< 2 yrs** | 92 | 1.80% | 25 | 0.80% | 10 | 0.80% |
| **2-<5 yrs** | 40 | 0.80% | 4 | 0.10% | 2 | 0.20% |
| **5-<15 yrs** | 14 | 0.30% | 4 | 0.10% | 1 | 0.10% |
| **15-<45 yrs** | 9 | 0.20% | 1 | 0.00% | 2 | 0.20% |
| **45-<60 yrs** | 5 | 0.10% | 0 | 0.00% | 0 | 0.00% |
| **>60 yrs** | 5 | 0.10% | 2 | 0.10% | 0 | 0.00% |
| **ADINOVIRUS** | |  |  |  |  |  |
| **< 2 yrs** | 54 | 1.00% | 5 | 0.20% | 6 | 0.50% |
| **2-<5 yrs** | 36 | 0.70% | 1 | 0.00% | 2 | 0.20% |
| **5-<15 yrs** | 41 | 0.80% | 9 | 0.30% | 1 | 0.10% |
| **15-<45 yrs** | 16 | 0.30% | 1 | 0.00% | 0 | 0.00% |
| **45-<60 yrs** | 2 | 0.00% | 1 | 0.00% | 0 | 0.00% |
| **>60 yrs** | 1 | 0.00% | 1 | 0.00% | 0 | 0.00% |
| **RHINOVIRUS** | |  |  |  |  |  |
| **< 2 yrs** | 73 | 1.40% | 4 | 0.10% | 4 | 0.30% |
| **2-<5 yrs** | 29 | 0.60% | 3 | 0.10% | 1 | 0.10% |
| **5-<15 yrs** | 23 | 0.40% | 2 | 0.10% | 1 | 0.10% |
| **15-<45 yrs** | 24 | 0.50% | 2 | 0.10% | 5 | 0.40% |
| **45-<60 yrs** | 9 | 0.20% | 0 | 0.00% | 0 | 0.00% |
| **>60 yrs** | 6 | 0.10% | 1 | 0.00% | 2 | 0.20% |
| **SARS CoV-2** | |  |  |  |  |  |
| **< 2 yrs** | NA | NA | 2 | 0.00% | 0 | 0.00% |
| **2-<5 yrs** | NA | NA | 5 | 0.10% | 1 | 0.10% |
| **5-<15 yrs** | NA | NA | 6 | 0.10% | 4 | 0.30% |
| **15-<45 yrs** | NA | NA | 207 | 4.00% | 33 | 2.70% |
| **45-<60 yrs** | NA | NA | 150 | 2.90% | 28 | 2.30% |
| **>60 yrs** | NA | NA | 153 | 2.90% | 38 | 3.10% |

**Supplementary t**able 2 Timeline of restrictions in India during COVID-19 pandemic

| S. No. | Phase | Start | End |
| --- | --- | --- | --- |
| 1 | Pre-lockdown (PL) | 1-Jan-20 | 24-Mar-20 |
| 2 | Lockdown 1.0 (LD1.0) | 25-Mar-20 | 14-Apr-20 |
| 3 | Lockdown 2.0 (LD2.0) | 15-Apr-20 | 3-May-20 |
| 4 | Lockdown 3.0 (LD3.0) | 4-May-20 | 17-May-20 |
| 5 | Lockdown 4.0 (LD4.0) | 18-May-20 | 31-May-20 |
| 6 | Unlock 1.0 (UL1.0) | 1-Jun-20 | 30-Jun-20 |
| 7 | Unlock 2.0 (UL2.0) | 1-Jul-20 | 31-Jul-20 |
| 8 | Unlock 3.0 (UL3.0) | 1-Aug-20 | 31-Aug-20 |
| 9 | Unlock 4.0 (UL4.0) | 1-Sep-20 | 30-Sep-20 |
| 10 | Unlock 5.0 (UL5.0) | 1-Oct-20 | 31-Oct-20 |
| 11 | Unlock 6.0 (UL6.0) | 1-Nov-20 | 30-Nov-20 |
| 12 | Lockdown in 2021 | 5-Apr-21 | 15-Jun-21 |

**Supplementary table 3.** Distribution of ARI and SARI positivity as per virus type.

| **ARI** | | **positivity %** | | | | | | | | |
| --- | --- | --- | --- | --- | --- | --- | --- | --- | --- | --- |
| **Age in Year** | **Inf A H1N1pdm09** | **Inf A H3N2** | **Inf B Vic** | **Inf B Yama** | **RSV [A+B]** | **PIV [All]** | **hMPV** | **Adeno virus** | **Rhino virus** | **nCov2019** |
| **<2 yrs** | 4.7% | 6.5% | 3.6% | 0.0% | 43.8% | 4.1% | 2.4% | 3.6% | 2.4% | 0.6% |
| **2-4 yrs** | 5.6% | 6.6% | 6.6% | 1.3% | 17.2% | 4.6% | 1.0% | 2.6% | 2.0% | 1.3% |
| **5-14 yrs** | 4.2% | 5.6% | 6.3% | 1.1% | 3.9% | 1.1% | 1.2% | 3.2% | 0.5% | 1.1% |
| **15-44** | 3.5% | 1.3% | 1.3% | 0.3% | 1.9% | 0.5% | 0.5% | 1.2% | 0.8% | 7.6% |
| **45-59 yrs** | 2.5% | 1.9% | 0.0% | 1.2% | 0.6% | 0.6% | 0.3% | 0.0% | 0.6% | 14.2% |
| **>=60 yrs** | 2.6% | 2.2% | 0.0% | 0.0% | 0.4% | 0.4% | 0.4% | 0.0% | 0.0% | 17.2% |
|  |  |  |  |  |  |  |  |  |  |  |
|  |  |  |  |  |  |  |  |  |  |  |
|  |  |  |  |  |  |  |  |  |  |  |
|  |  |  |  |  |  |  |  |  |  |  |
| **SARI** | | **Positivity %** | | | | | | | | |
| **Age in Year** | **Inf A H1N1pdm09** | **Inf A H3N2** | **Inf B Vic** | **Inf B Yama** | **RSV [A+B]** | **PIV [All]** | **hMPV** | **Adeno virus** | **Rhino virus** | **nCov2019** |
| **<2 yrs** | 3.3% | 0.7% | 1.8% | 0.2% | 22.8% | 6.8% | 4.6% | 3.5% | 4.3% | 0.1% |
| **2-4 yrs** | 7.8% | 2.7% | 3.4% | 0.7% | 15.0% | 4.4% | 5.7% | 4.2% | 3.7% | 0.3% |
| **5-14 yrs** | 6.3% | 2.2% | 5.3% | 1.0% | 3.6% | 1.8% | 1.9% | 4.5% | 3.1% | 0.5% |
| **15-44** | 5.7% | 0.9% | 1.0% | 0.1% | 0.5% | 0.4% | 0.2% | 0.1% | 1.5% | 10.3% |
| **45-59 yrs** | 6.0% | 0.3% | 0.0% | 0.0% | 0.9% | 0.3% | 0.6% | 0.3% | 0.8% | 14.9% |
| **>=60 yrs** | 2.8% | 0.5% | 0.2% | 0.9% | 1.0% | 0.5% | 0.6% | 0.2% | 0.7% | 12.3% |
